# Spontaneous Bio-Recycling: Recovering Bioactive Molecules Through Endogenous Microbial Maceration of Hemp Residues

**DOI:** 10.3390/microorganisms13020455

**Published:** 2025-02-19

**Authors:** Leonardo Donati, Angela Conti, Debora Casagrande Pierantoni, Chiara Ruspi, Martina Cerri, Carla Emiliani, Gianluigi Cardinali, Laura Corte

**Affiliations:** 1 Department of Chemistry, Biology and Biotechnology, University of Perugia, 06126 Perugia, Italy; leonardodonati_1993@libero.it (L.D.); carla.emiliani@unipg.it (C.E.); 2 Department of Pharmaceutical Sciences, University of Perugia, 06126 Perugia, Italy; angela.conti@unipg.it (A.C.); debora.casagrandepierantoni@unipg.it (D.C.P.); chiara.ruspi@dottorandi.unipg.it (C.R.); 3 Department of Agricultural, Food and Environmental Sciences, University of Perugia, 06126 Perugia, Italy; martina.cerri@unipg.it; 4 Centro di Eccellenza Materiali Innovativi Nanostrutturati (CEMIN), University of Perugia, 06126 Perugia, Italy

**Keywords:** *Cannabis sativa* L., microbiota, sustainability, maceration, metabarcoding, extracts, green economy

## Abstract

Biomass residues represent a major issue for industries. On the other hand, residues enclosed major classes of bioactive compounds that could be extracted and used across various fields. This study aimed to elucidate the role of the endogenous microbial community in the lignocellulosic degradation of hemp residues for biorefineries or other industrial processes, simultaneously characterizing the composition of three extracts recovered at different stages of maceration. The process was examined from different perspectives: plant tissue degradation and microbial dynamics were monitored using histological, cultural-dependent, and independent analysis. Extracts were characterized through FTIR, NMR, and SDS-PAGE analyses, also evaluating their potential as biostimulants for microbial growth. Results revealed that the composition of the endogenous prokaryotic community remained stable during the maceration period, while fluctuations in various fungal genera were observed. The taxonomical composition of hemp residues at different stages may account for the increased accumulation of amide-containing compounds, sugars, and other metabolites detected in long-term bioconversion. Finally, the extracts recovered from the microbial degradation of hemp residues were able to support the growth of the yeast *Cryptococcus phenolicus* and the bacterium *Peribacillus simplex* as the sole source of nitrogen, paving the way for their potential use as biostimulants.

## 1. Introduction

It was estimated that the amount of biomass waste generated each year is in the order of 140 Gt [1], with an annual increase at a rate of 5–10% [2]. Those numbers are generated from residues of manure and animal carcasses (animal waste), corn stalks, sugar cane bagasse, drops and culls from fruits and vegetables, pruning (crop waste), pesticides, insecticides, herbicides, and food processing waste [3]. Concerns about human health and environmental pollution pushed the research into the development of innovative systems in waste management and treatment technologies. One possible solution could be the use of biowastes as animal feeds and fertilizers. Nonetheless, most agricultural waste has macro- and micronutrients, as well as bioactive compounds that could have a high added value after agricultural waste valorization processes [4]. A recent study [5] demonstrated that processing low-valued feed-stocks produces beneficial transformations to the raw material such as improving protein quality, degrading lignin, and enhancing the utilization of dietary fibre for energy. Researchers concluded that upcycling existing “low-value” agro-industrial byproducts and food waste for animal feed could significantly recover and recycle waste nutrients within the food supply chain, together with other advantageous effects regarding environment protection. Methods to valorize waste may include the use of the pyrolysis of wood/biomass for biofuels, catalytic chemistry, thermochemical conversion, and biochemical technologies to convert biomass into value-added products [6]. The same considerations could be made for the 275,000 tonnes of industrial hemp that were estimated to be produced globally in 2019 [7]. In Europe, for example, the hemp growing area and production have increased from 20,540 ha to 33,020 ha and from 97,130 tonnes to 179,020 tonnes, respectively, in the period 2015–2022 [8]. Together with the increasing market potential, the hemp industry has to manage the main harvested products that account for a waste per plant total that measures approximately 74.6 g of fresh or 22.1 g of dry weight [9]. Numerous possible utilizations of hemp byproducts have been developed to reduce the impact of agro-industrial waste and generate economically feasible green resources [10]. One of the main applications of hemp byproducts is their use as a biofuel feedstock; in fact, hemp is more profitable than competing crops for producing ethanol and it has a much higher conversion rate than average, which is useful in the production of biodiesel [11]. Other researchers have attempted to turn these hemp byproducts into components in food applications [12,13,14,15], such as feedstuff for inclusion in livestock diets [16]. The structure of raw material could represent a limitation to the process of conversion. Hemp fibres in fact consist of celluloses, hemicellulose, and lignin, with a combination of crystalline (a structure of the cellulose) and amorphous regions that complicate transformation [17]. Thus, to produce such valuable products, the process of biomass conversion needs to be supplemented with an efficient pretreatment in order to enhance downstream processes [18]. Since hemp is characterized by a robust structure and complex physicochemical composition, different approaches have been studied, from the classical chemical methods to the physical alternatives such as ultrasound [19] and electron beam irradiation [20]. Among these protocols, organic solvent pretreatment shows significant yields in the recovery of the lignocellulosic biomass into its main constituents but is more expensive due to the additional cost of reagents [21], other than posing environmental issues due to acid waste disposal [22]. A safer method, which is eco-friendly and does not require energy consumption, is the employment of microorganisms. It was also demonstrated that the combination of white-rot fungi and the chemical pretreatment of industrial hemp woody cores improved enzymatic saccharification [23]. Such a method could also be useful to produce intermediates rich in bioactive compounds. Many studies have demonstrated that it is possible to extract nutritional components from plants such as cereals [24,25,26], tomato residues [27], olive mill stone waste, and walnut shells [28] through solid-state fermentation [29]. In line with the idea of biorefineries, hemp pretreatment with microorganisms could both produce high-value chemicals and loosen the matrix for further processing. In this context, our aim was to follow the dynamic of the endogenous microbial population and its ability to extract bioactive compounds suitable as ingredients in food and pharmaceutical products [30,31]. We described the species involved in the degradation, the timing of the different phases, and the molecules that could be extracted from hemp residues.

## 2. Materials and Methods

### 2.1. Hemp Residues

Waste of *Cannabis sativa* L. was kindly supplied by JJ Farm Società Agricola Semplice [Castiglione del Lago (Pg), Umbria, Italy]. The study was performed with the Strawberry variety, cultivated in outdoor natural conditions without any addition of chemicals. The plants were harvested manually and immediately dried in closed sheds in the dark at 22 °C with 45% humidity for 15 days. The flowers were then separated from leaves and stems using a mechanical system, and the waste (stems, leaves, and floral residues) was collected in sealed plastic bags and stored in the dark, at room temperature, until our analysis.

### 2.2. The Maceration Process

Waste material was weighted to reach 100 g of dry material and gently crushed in a pestle to a small size to facilitate the extraction procedure. The residue was covered with 800 mL of deionized sterile water (ddsH_2_O) in a closed sterile glass jar. Jars were incubated at 25 °C for three months, with their contents stirred thoroughly twice a week. Three biological replicas were prepared, each tested in triplicate. Samples were collected at the beginning of maceration (t0), every week up to two months (from t1 to t8) and at the end of the three months of maceration (t12).

### 2.3. The Evolution of the Microbial Community During the Hemp Maceration Process

The evolution of the microbial community during the whole maceration process was monitored by viable plate counts and by evaluating the taxonomic composition through *ITS* and *16S rRNA* gene metabarcoding. In parallel, light microscope analysis was applied to evaluate the changes that occurred in the plant tissues during the process.

#### 2.3.1. Viable Microbial Cell Count

The viable count was carried out considering, separately, total bacteria, bacteria of the *Lactobacillus genus*, and yeasts, using the following media: BHI (HiMedia Laboratories, Maharashtra, India) 37 g L^−1^, MRS (Merck, Milano, Italia) 51.25 g L^−1^, and YEPD (HiMedia Laboratories, Maharashtra, India) Peptone 5 g L^−1^; Yeast extract 5 g L^−1^; Glucose 20 g L^−1^ + Chloramphenicol (1% *v*/*v*; 0.5 g L^−1^). Each sample was plated in triplicate. The plates were incubated at 25 °C for 3 days. The count of total bacteria and yeasts was carried out both in aerobic and microaerophilic conditions while that of Lactobacilli was carried out only in microaerophilic conditions. Data were expressed as CFU mL^−1^.

#### 2.3.2. Taxonomic Composition with *16S rRNA* Gene and *ITS* Metabarcoding

##### DNA Extraction

Samples described in Section 2.2 were also analyzed through a metabarcoding approach. All the replicates were homogenized for 5 min at 70 rpm and then centrifuged for 5 min at 4500 rpm. The supernatant was discarded and the pellet was resuspended with 800 µL of CD1 (© QIAGEN, Venlo, The Netherlands) provided by DNeasy PowerSoil Pro Kit. DNA extraction was then performed according to the manufacturer’s instructions given with the above-mentioned kit.

##### PCR Amplification and Oxford Nanopore (ONT) Sequencing

Metagenomic DNA was used as a template for the PCR amplification of the standard barcode regions currently employed in microbial taxonomy: *16S rRNA* gene for prokaryotes and *ITS* together with *LSU D1/D2* for Eukaryotes. The whole *16S rRNA* gene was amplified with the primer pair 8F (5′-AGAGTTTGATCCTGGCTCAG) [32] and 1492R (5′-GGTTACCTTGTTACGACTT) [33].

Marker loci *ITS1, 5.8S, ITS2*, and the D1/D2 domain of *26S* subunit were amplified in contiguity using the primers ITS1 (5′-TCCGTAGGTGAACCTGCGG)/NL4 (5′-GGTCCGTGTTTCAAGACGG) [34].

Platinum™ SuperFi II PCR Master Mix (Invitrogen™, Camarillo, CA, USA) was chosen to carry out the amplifications because of its Platinum SuperFi II Buffer that enables universal primer annealing. For both barcodes, the PCR protocol was carried out as follows: initial denaturation at 98 °C for 30 s, 30 amplification cycles (at 98 °C for 30 s, 60 °C for 1 min, and 72 °C for 45 s) and a final extension at 72 °C for 5 min. Amplicons were checked on 1% Agarose gel. ONT sequencing was carried out following the procedure described in the Ligation Sequencing Kit SQK-LSK109 protocol (Oxford Nanopore, Oxford, UK). *ITS* and *16S rDNA* Minion sequences are stored in the SRA archive with the BioProject ID PRJNA1217596.

##### Sequence Analysis

FASTA5 produced with MinION (Oxford Nanopore, Oxford, UK) was basecalled with Guppy (version 6.4.6) on a supported NVIDIA RTX-A 4000 GPU (Santa Clara, CA, USA). The sequence analysis pipeline worked in a conda environment built in Ubuntu. Filtering processes of raw reads were carried out using the function seqtk, which removed sequences below 400 bp and greater than 1800 bp. Filtered reads were merged into one file that was used as input for the alignment program minimap2 (version 2.24). UNITE and SILVA were used as reference databases. The taxonomic abundance was calculated with the function “trans_abund” (microeco, R version 4.4.1). Package ggplot2 (version 3.5, Wickham, 2016) was used to create a bar plot.

#### 2.3.3. Light Microscopy and Image Analysis

Samples were fixed in 3% (*w*/*v*) glutaraldehyde in 0.075 M cacodylate buffer, with a pH of 7.2, for 10 h; they were washed three times for 7 min in 0.075 M cacodylate buffer at a pH of 7.2 and finally post-fixed in 1% (*w*/*v*) OsO4 in the same buffer for 1 h. After dehydration in increasing concentrations of ethanol, samples were embedded in epoxy resin (Epon, 2-dodecenylsuccinic anhydride, and methylnadic anhydride mixture) [35]. Semi-thin sections (1–2 μm), obtained with an ultramicrotome (OmU2, Reichert, Heidelberg, Germany) equipped with a glass blade, were stained with toluidine blue 0.1% (*w*/*v*) and observed under a light microscope (BX53; Olympus, Tokyo, Japan) and through the software CellSens (version 3.1.1 Olympus, Tokyo, Japan).

### 2.4. Hemp Extracts (HWEs)

At the end of the short- (72 h) and long-term maceration process (one and two months), three different hemp extracts (HEs) were obtained (Table 1), as already detailed in our previous paper by Donati and colleagues [36].

### 2.5. Hemp Extracts (HEs) Chemo-Physical and Biological Characterization

#### 2.5.1. NMR Analysis

NMR measurements were performed according to Donati and colleagues [36].

#### 2.5.2. FTIR Analysis

One milligram of each HE was resuspended in 1 mL of HPLC (High-Performance Liquid Chromatography) water for FTIR analysis. A 105 μL volume was then sampled for three independent FTIR readings (35 μL mL each), according to the technique suggested by Essendoubi et al. [37]. Each sample was analyzed in triplicate. FTIR measurements were performed in transmission mode, recording spectra in the range between 3800 and 500 cm^−1^, with a 4 cm^−1^ spectral resolution and setting 256 scans per sample. Quality testing, baseline correction, background subtraction, vector normalization, and the calculation of the peak integral areas were carried out using the software OPUS, version 6.5 (Bruker Optics GmbH, Ettlingen, Germany). The integral area analysis was performed by categorizing spectra into specific regions: (1) 3500–2800 cm^−1^, (2) 1700–1500 cm^−1^, (3) 1450–1300 cm^−1^, and (4) 1200–900 cm^−1^. The data were subjected to an analysis of variance (pairwise ANOVA test) in an R environment (https://cran.r-project.org/, accessed on 1 February 2024). The significance level was established at *p* ≤ 0.05 using Tukey’s post hoc test.

#### 2.5.3. SDS-PAGE

SDS-PAGE was carried out according to Laemmli’s method [38], using a 12% (*v*/*w*) acrylamide resolving gel and a 4% acrylamide stacking gel. Samples were prepared by mixing them with 5× sample buffer (0.5 M Tris–HCl buffer pH 6.8, containing 10% (*w*/*v*) SDS, 50% (*v*/*v*) glycerol, 0.01% (*w*/*v*) bromophenol blue and 125 mM dithiothreitol, DTT) and incubated at 95 °C for 5 min; after that, the samples were immediately left on ice for 5 min. Appropriate volumes were then loaded into the gel, corresponding to 40 µg of proteins, and subjected to a previous electrophoretic run at 20 mA for the first 30 min and 40 mA for 1 h. The stroke was carried out using the known molecular weight standard as a reference. The running buffer used (electrode buffer) was Tris 0.025 M/glycine 0.192 M containing 1% SDS (*w*/*v*).

#### 2.5.4. Growth Assay with HEs as Source of Nutrients

To verify whether and to what extent the three HEs could be used as biostimulants or food supplements, a growth test was performed on a panel of nine strains, five bacterial strains, and four yeast strains (Table 2), providing the HEs as the sole nitrogen source. Microorganisms were chosen for their role in the environment: bacterial strains belong to species classified as Plant Growth Promoters [39,40,41,42] in the literature, while yeast strains are related to biocontrol activities [43,44,45,46,47].

All strains were provided by the CMC collection of CEMIN (Centre of Excellence for Innovative Nanostructured Materials for Chemical Physical and Biomedical Applications—University of Perugia). Pre-inocula were prepared for each strain by picking a single colony from Agar plates and inoculating it in YNB (Yeast Nitrogen Base, HIMEDIA, 6.75 g L^−1^) supplemented with 2% glucose for yeasts or M9 (3.0 g L^−1^ KH_2_PO_4_, 6.0 g L^−1^ Na_2_HPO_4_, 1 g L^−1^ NH_4_Cl, 0.5 g L^−1^ NaCl, 0.003 g L^−1^ CaCl_2_.) supplemented with 2% glucose for bacteria. Liquid cultures were incubated for 16 hrs under agitation (120 rpm min^−1^). Cell density was determined using a spectrophotometer at λ 600 nm (OD_600_) and this was used to calibrate the inoculum for the assay at OD_600_ = 0.1. Test conditions were represented by a medium composed of hemp extracts at different concentrations (from 1 to 0.125 mg mL^−1^) supplemented with 2% glucose. The control test was represented by the growth of each strain into YNB 2% glucose for yeast and M9 2% glucose for bacteria. The growth tests were carried out in 96-well plates, and incubation lasted for 24 h in a Tecan Infinite spectrophotometer. Measurements were acquired every 5 min at λ 600 nm. All experiments were carried out with three biological and technical replicates. The effect of hemp extracts was evaluated by comparing the growth in standard experimental conditions. This measure was reported as the fold change by dividing the experimental optical density by the optical density obtained in standard control, as follows:

FC = Experimental optical density/standard optical density

Statistical analysis was performed with a one-tailed paired *t*-test (MS Excel) to evaluate the significance of the comparison between the standard and experimental conditions.

## 3. Results

### 3.1. Histological Analysis to Assess the Level of Hemp Degradation

**Figure 1:**
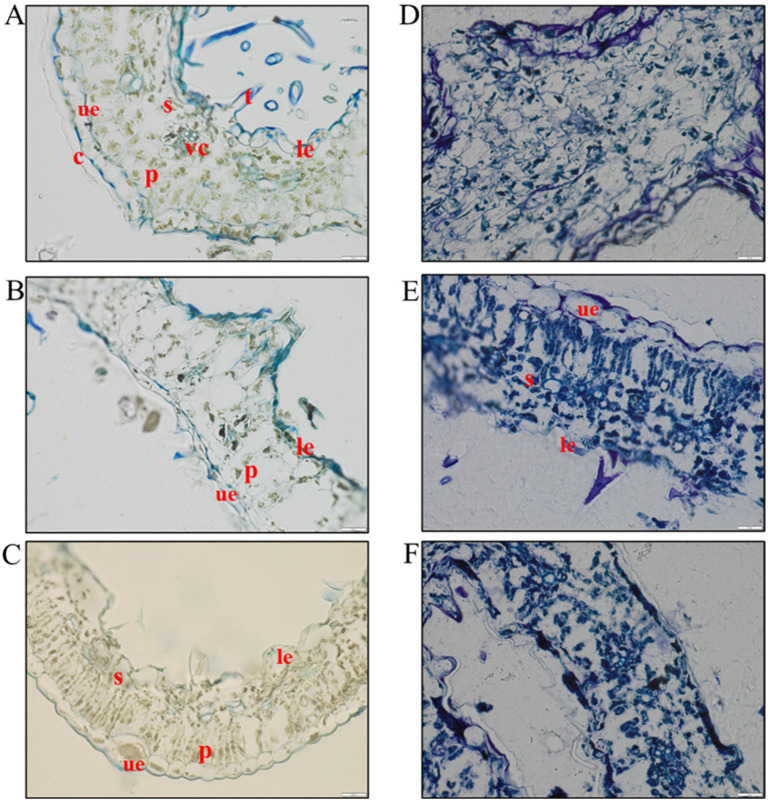
Leaf sections stained with toluidine blue at different sampling points. (**A**) Leaf at T0, without signs of the degradation process. (**B**) Samples after one week. (**C**) Samples after 3 weeks, where the structure is still maintained. (**D**) Samples after 5 weeks, where chloroplasts showed degradation. (**E**) Samples after 8 weeks, where neither cell walls nor membranes were appreciable. (**F**) Samples after 12 weeks, with leaf structure completely damaged. c = cuticle, ue = upper epidermis, p = palisade-like parenchyma with chloroplasts, vc = vascular tissue and cribrous tissue, s = spongy parenchyma, le = lower epidermis, and t = trichomes. In white Scale bar: 20 µm.

To follow the maceration process and verify the possible contribution of native microorganisms, fibre samples were collected weekly for two months (t0, t1, t2, t3, t4, t5, t6, t7, and t8). One more sample was taken after three months of maceration (t12). As expected, samples appeared damaged due to the preliminary comminution of the residues, which made it difficult to distinguish the different plant organs and thus obtain perfectly transversal sections. The results of the histological analysis were reported in Figure 1. At the beginning of the maceration process (t0), it is possible to distinguish the plant tissues typical of leaves: an upper epidermis covered by a cuticle, a palisade-like parenchyma with chloroplasts, vascular tissue with lignified cells (light blue), and cribrous tissue, a spongy parenchyma with numerous intercellular spaces, a lower epidermis, and trichomes (Figure 1A). After one and three weeks of maceration (t1 and t3), cell walls and chloroplasts were still evident, as well as cells of both epidermises (Figure 1B,C). After five weeks (t5), cell walls were still well defined. At this stage, as we could not obtain transversal sections due to the sample characteristics, it was difficult to identify palisade and spongy tissues. At the same time, chloroplasts and cytoplasm started to show the first signs of degradation (Figure 1D). At the end of the two months of maceration (t8) and, above all, at t12 (three months), cell walls and membranes appeared damaged and digested, and chloroplasts appeared less sharp (Figure 1E,F).

### 3.2. Evaluating Microbial Dynamics During Hemp Degradation

To follow the variation in the whole microbial community during the degradation process, cultural and metabarcoding analyses were carried out on the macerated fractions collected at the different times of sampling from t1 to t12.

#### 3.2.1. Cultural-Dependent Analysis

**Figure 2:**
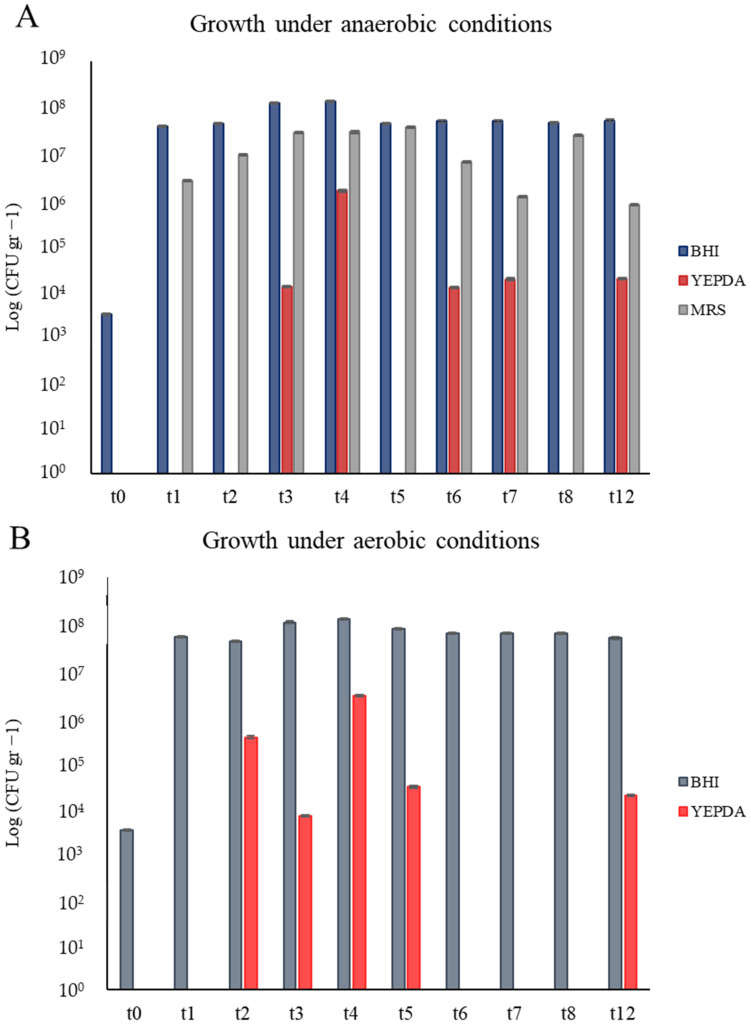
Results from viable counts into two different growth conditions. The y-axis reports the log value of the cellular density expressed as CFU gr^−1^. Standard deviation is also indicated using the error bars on the top of each bar. (**A**) Anaerobic conditions: cellular density of prokaryotes in BHI medium (blue bars), eukaryotes in YEPD with Chloramphenicol medium (red bars), and microbes of the *Lactobacillus* genus in MRS medium (grey bars). (**B**) Aerobic conditions: cellular density of prokaryotes on BHI medium (bluish grey bars) and eukaryotes in YEPD with Chloramphenicol medium (red bars).

For a comprehensive study of the evolution of the microbial community over the maceration time, all samples were plated into three different media to focus on prokaryotes, eukaryotes, and microaerophilic bacteria separately. As already detailed in the Materials and Methods section, BHI and YEPD media were incubated in both aerobic and anaerobic conditions, while the MRS medium was only incubated in anaerobiosis. The first piece of evidence (Figure 2) was that anaerobic and aerobic conditions gave similar results for the growth of prokaryotes but not for the eukaryotes. Fungi started to be detectable on YEPD medium from samples after 15 days (t2, Figure 2B) of maceration in aerobic conditions, while this was the case only after 21 days (t3, Figure 2A) for anaerobic conditions. The same results were obtained on YEPD with samples at 35, 42, and 49 days of maceration (t5, t6, and t7). Fungi at t5 were countable when incubated in aerobic (Figure 2B) rather than in anaerobic conditions (Figure 2A). On the contrary, t6 and t7 were countable for the test in anaerobiosis (Figure 2A) but uncountable in aerobiosis (Figure 2B). Finally, in both conditions, the YEPD plates were not countable for samples after two months of maceration (t8), due to a low concentration of cells, while at t12 (after three months), the concentration exceeded 10^4^ CFU mL^−1^.

On the contrary, prokaryotes showed a stable dynamic both in anaerobiosis and aerobiosis, with values of concentration in the order of 10^7^ CFU mL^−1^, and a peak between t3 and t4 of around 10^8^ CFU mL^−1^. Similarly, microaerophilic bacteria on MRS fluctuated between 10^6^ and 10^7^ CFU mL^−1^, down to 10^5^ CFU mL^−1^ at the end of the process (t12).

Notably, each of the three microbial classes analyzed underwent specific growth at specific times during the maceration process. In terms of microbial growth on BHI medium, there was a rapid increase from t0 to t1, with counts rising from 10^3^ to 10^7^ ca CFU mL^−1^. Microaerophilic bacteria, not detectable at t0, jumped to 10^6^ CFU mL^−1^ after one week of maceration (t1). As previously reported, fungi only became countable after 15 days of maceration (t2).

#### 3.2.2. Metabarcoding

**Figure 3:**
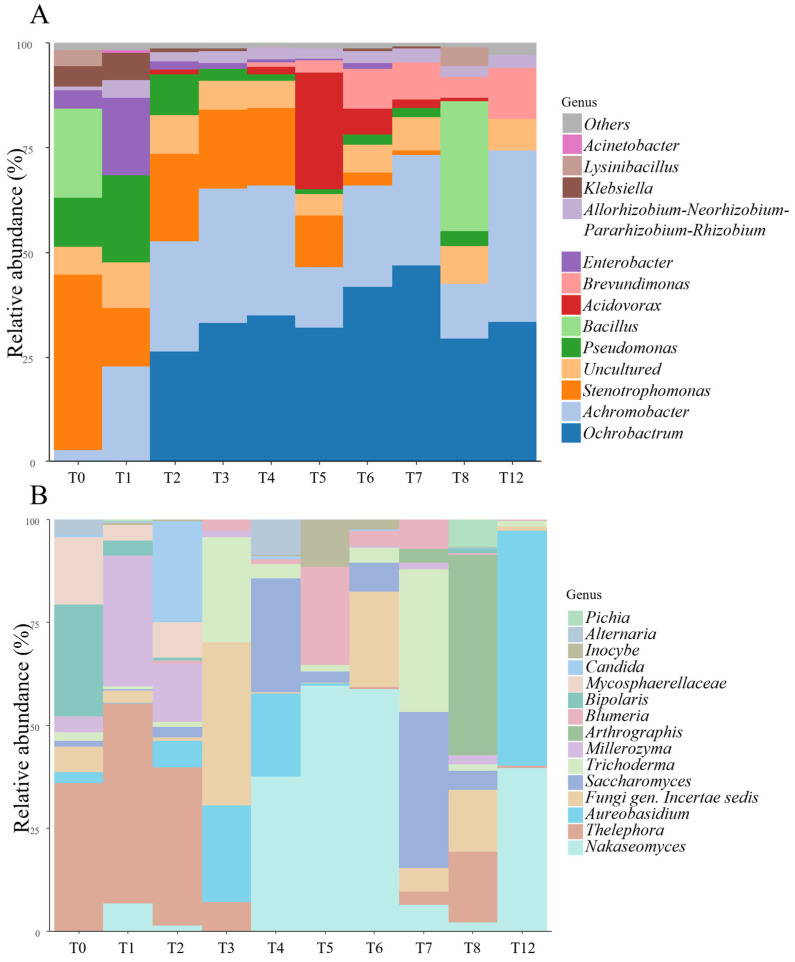
Bar plot describing microbial dynamics through the maceration process. For each fraction (*x*-axis), the abundances at the genus level are vertically arranged in blocks of different colours and widths. Colours indicate different genera; conversely, the same colour is used to define the same genus. Colour codes are described in the legend on the right. The width indicates the relative abundances—the wider the block, the more abundant the genus. (**A**) Relative abundance calculated on *16S rRNA* gene sequences to describe prokaryotes dynamic. (**B**) Relative abundance calculated on *ITS* sequences to follow the eukaryotic dynamic.

The taxonomic analysis of the microbial population confirmed the findings from the microbial community growth analysis, showing that prokaryotes remained relatively stable over time, whereas the fungal community exhibited significant variation (Figure 3B). From a bacterial perspective, there was a substantial shift in the community between t0 and t2 (Figure 3A). In the raw material (t0), in fact, 40% of the *16S rRNA* gene reads were identified as belonging to species of the genus *Stenotrophomonas*, while in t1, the most abundant genus was *Achromobacter*, accounting for 30% of the reads. The second most abundant genus in t1 was *Pseudomonas*, which peaked at 20% of the sequenced reads. By t2, there was a noted decrease in the number of reads attributed to the *Stenotrophomonas* genus, accompanied by a rapid increase in the abundance of *Ochrobactrum*. From t2 to t12, *Ochrobactrum* and *Achromobacter* were the most abundant prokaryotic genera identified via *16S rRNA* gene sequencing.

Similarly, the group *Allorhizobium*–*Neorhizobium*–*Pararhizobium*–*Rhizobium* remained relatively constant over time, with about 2% of the total *16S rRNA* gene sequences assigned to them. Despite the stability observed in the prokaryotic community, t4 saw the emergence of *Brevundimonas*, which showed a steady increase in abundance, reaching 22% of the total reads by t12. At the same time (t4), there was an increase in reads for the genera *Nakasomyces* and *Saccharomyces* (Figure 3B), corroborating the cultural analysis results that showed a peak in yeast concentration in this fraction.

Unlike the *16S rRNA* gene data, *ITS* metabarcoding revealed an uneven trend in the fungal community. For instance, *Nakasomyces* reads were slightly detected at t1, increased significantly between t4 and t6 (up to 60% at t5), dropped to 2% at t7 and t8, and then rose again at t12. Similarly, *Saccharomyces ITS* represented 30% of the reads at t4 and t7, while its average percentage in other fractions was around 3%. The same dynamic can be illustrated at different times by considering reads identified as belonging to the genus *Aureobasidium*. These were found after two weeks of maceration (t2), with their relative abundance increasing at t3 and t4, before becoming undetectable until t12. At t12, *Aureobasidium* represented the highest portion of the fungal community in terms of sequenced reads, with 56% of *ITS* sequences mapping to this genus. Whether the central period of the maceration process (from t3 to t6) was characterized by the presence of reads related to the order *Saccharomycetales*, the fungal community in the raw material was represented by the genus *Thelophora* and *Bipolaris*. It is important to underline the fact the fungal composition after two months of maceration (t8) was predominantly moulds such as *Arthrographis sp*. and species identified as *Fungi Incertae sedis*. By t12 (three months), only yeast (*Nakasomyces* sp.) and yeast-like (*Aureobasidium* sp.) organisms were identified.

To deepen the study of the microbial dynamic across time, the level of similarity among the different samples was computed using the hierarchical clustering method (Figure S1). This analysis confirmed that the macerates can be grouped into three clusters based on the maceration time: early maceration (t0, t1, and t2), medium maceration (t4, t5, and t6) and late maceration (t7 and t8). As already reported, there is a considerable difference in the fungal community among the three groups, while the bacterial community remains almost invariable. Notably, the similarity found between the t3 fraction (3 weeks of maceration) and the late maceration group is likely attributable to the peaks of *Trichoderma* and *Fungi Incertae sedis* detected in both t3 and t7. Likewise, the placement of t12 within the same cluster as t4 is significant, with *Saccharomyces* and *Brevundimonas* being the common genera between these two fractions.

### 3.3. Characterization of the Hemp Extracts

With the aim of isolating bioactive compounds from the hemp degradation process, extracts at three different times were considered: S14 represents the short-term maceration considering that it was taken three days from the beginning of the experiments, while S9 and S21 constitute two examples of long-term maceration because they were extracted, respectively, after one and two months of degradation. To describe their composition NMR, FTIR analysis, and SDS-PAGE were carried out.

#### 3.3.1. Chemical Characterization with NMR and FTIR

**Figure 4:**
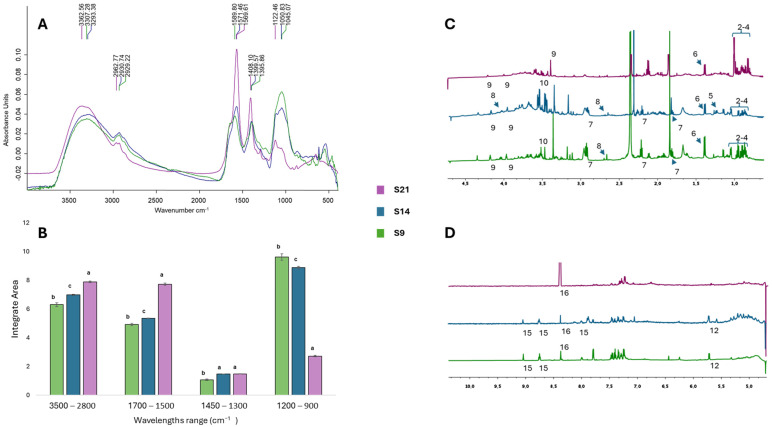
NMR and FTIR characterization of S14 (blue), S9 (green), and S21 (purple) hemp extract samples. FTIR spectra: (**A**) Average IR spectra with the major peaks as-signed; (**B**) mean integral areas calculated in the ranges between 3500 and 2800 cm^−1^, 1800 and 1700 cm^−1^, 1450 and 1300 cm^−1^, and 1200 and 900 cm^−1^. Data are presented as means ± SD (*n* = 3). Significant differences are represented with different letters, according to the ANOVA pairwise test (*p* ≤ 0.05). NMR spectra: (**C**) Hight field resonances for spectra of samples acquired in D_2_O using AU_WATERSC pulse program. Peak assignment is reported: ^2^ valine; ^3^ isoleucine; ^4^ leucine; ^6^ alanine; ^7^ GABA; ^8^ malic acid; ^9^ choline; ^10^ glycerol and its derivatives. (**D**) Low field resonances (10× tenfold-amplified) for spectra acquired in D_2_O using the AU_WATERSC pulse program. Peak assignment is reported: ^12^ sucrose; ^15^ trigonelline; ^16^ formic acid.

The biochemical profiles of S9, S14, and S21 hemp extracts were characterized through NMR and FTIR analyses (Figure 4). For the NMR results, tentative assignments are proposed through the comparison of the observed chemical shifts with those reported in the literature and/or in HMDB (Human Metabolome Database). FTIR peaks were assigned by comparison with those reported in the literature. A quantitative analysis of the integrated areas of the specific lipids, esters, proteins, and carbohydrate FTIR bands was presented. The area integrals were calculated from the primary FTIR spectra upon baseline correction and vector normalization.

The analysis of the FTIR spectra revealed that the duration of the endogenous microbial maceration of hemp residues significantly affected the composition of the extracts, which differed in absorption strengths and for the entire shift in the S21 spectrum.

Common peaks were recovered within the following ranges: 3500–2800 cm^−1^, 1700–1500 cm^−1^, 1450–1300 cm^−1^, and 1200–900 cm^−1^ (Figure 4A,B). In these regions, the intensity of the peaks was observed in the following order of adsorption strength S21 > S14 > S9, except for the carbohydrate region where the order of adsorption strength was reversed to S9 > S14 > S21 (Figure 4A).

The peaks between 3300 and 3400 cm^−1^ and 2910–2930 cm^−1^ were usually assigned to the O–H stretching of cellulose and hemicellulose and the C–H stretching in cellulose, while those in the absorbance range 1570–1590 cm^−1^ were attributed to the stretching vibrations of C=C bonds in aromatic rings of lignin [48,49].

In the ranges 3500–2800 cm^−1^ and 1700–1500 cm^−1^, the integrated area analysis revealed that the amplitude of the S21 peak was approximately 15% higher than that of S9 and S14 in the first region and 30% higher in the second region (Figure 4B).

All samples displayed absorption peaks in the range of 1450–1300 cm^−1^, which mainly arose from stretching and bending vibrations of methyl and methylene groups in proteins and carbohydrates [50].

Significant differences were also detected in the carbohydrate region (1200–900 cm^−1^), dominated by ring vibrations overlapped with stretching vibrations of (C–OH) side groups and the (C–O–C) glycosidic bond vibration [51]. In this region, the greatest variation was recorded for extract S9, obtained at the end of the first month of maceration, which displayed a peak area 14% larger than that of S14 and 70% larger than that of S21 (Figure 4B). The bands found between 1040 and 1050 cm^−1^ for S9 and S14 samples, shifted to 1122 cm^−1^ for S21, could be referred to the presence of rhamnogalacturonan, a typical polysaccharide of plant cell walls [51].

NMR data supported and complemented the results obtained with FTIR characterization (Figure 4C,D). NMR spectra revealed the presence of sucrose in all extracts (peak 12). This peak was only barely detectable in S21, which instead showed an accumulation of formic acid (peak 16), a secondary product of the anaerobic metabolism of microorganisms such as *Escherichia coli* that in the anaerobic fermentation pathway converts pyruvate into acetyl-CoA and formate, which can then be further metabolized into formic acid [52].

Finally, all extracts showed signals corresponding to amino acids such as valine, isoleucine, leucine, and alanine (peaks 2, 3, 4, and 6) and bioactive compounds, with patterns associated with gamma-aminobutyric acid (GABA) (peak 7) in all extracts, choline (peak 9) in S9 and S21 (Figure 4C), and traces of trigonelline (peak 15) in S14 and S9 (Figure 4D).

Taken together, these data reinforce the evidence already produced by the histological analysis (Figure 1). During the two months of maceration of the hemp residues, the endogenous microbial community played a major role in the degradation of plant cell walls, inducing a progressive increase in lignin content as the cellulose and hemicellulose were removed from the hemp fibres [53]. The metabolism of the endogenous microbial community also led to a progressive accumulation of polysaccharides and simple sugars, accompanied by the presence of amide-containing and bioactive compounds, underscoring the potential of this approach for extracting valuable compounds from hemp residues.

#### 3.3.2. Biochemical Characterization with SDS-PAGE

**Figure 5:**
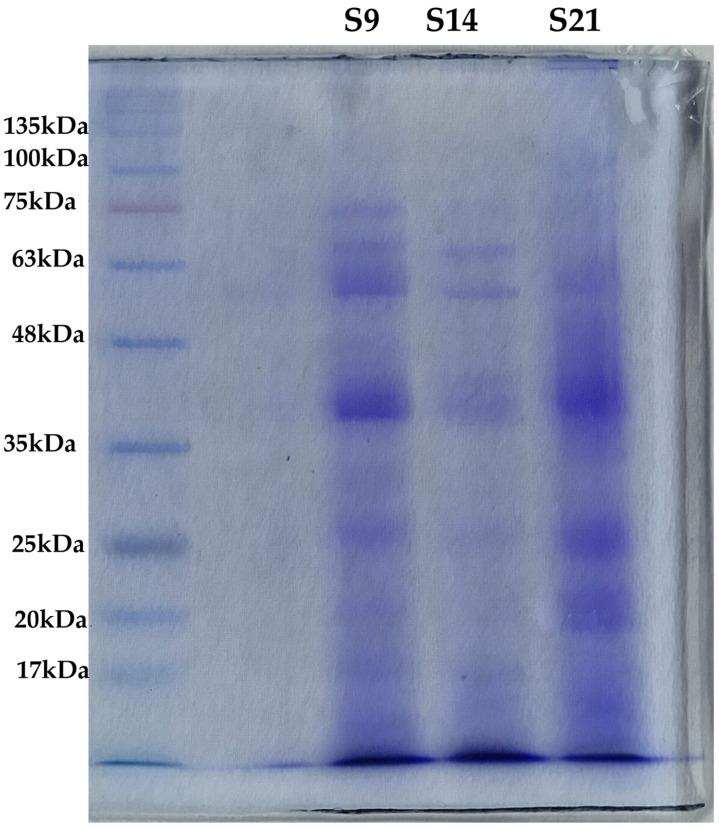
SDS-PAGE to evaluate protein content in the three extracts. The leftmost lane contains the molecular marker with a range of weights comprised between 135 kDa and 17 kDa. From left to right, samples were loaded as follows: S14, S9, and S21. Bands are clearly distinguishable in S14 and S9, while S21 is characterized by a smear of between 75 kDa and 35 kDa. The intensity of the band is comparable to the protein concentration in the sample.

The SDS-PAGE profiles of HEs are presented in Figure 5. This analysis validated what has already been hypothesized by the analysis of the spectral data (Figure 4) on the increase in the amino acid contents of HEs as the maceration time increases. In fact, the smear shown by the only S21 extract is a clear signal of protein degradation, confirmed by the fading of the bands above 63 kDa and the increase in intensity of the band at 20 kDa, almost absent in both S9 and S14. Interestingly, the band at 37 kDa, slightly visible in S14, intensified in S9 until it spread into S21, further supporting the degradation process. Considering the protein profile of S14 and S9, there are not many differences in terms of bands, but rather in their different intensity. This is consistent with the results presented by Hadnađev and colleagues [54], who found that at pH values between 5 and 7, hemp proteins showed minimal solubility, while in alkaline or strongly acidic solutions, the level of solubility increased. Given the pH of the three HEs obtained, equal to 5.74 for S14 and around 8 for S9 and S21, the lower presence of proteins found in S14 could be attributable to its lower solubilization.

### 3.4. Hemp Extracts as Nutrients

**Figure 6:**
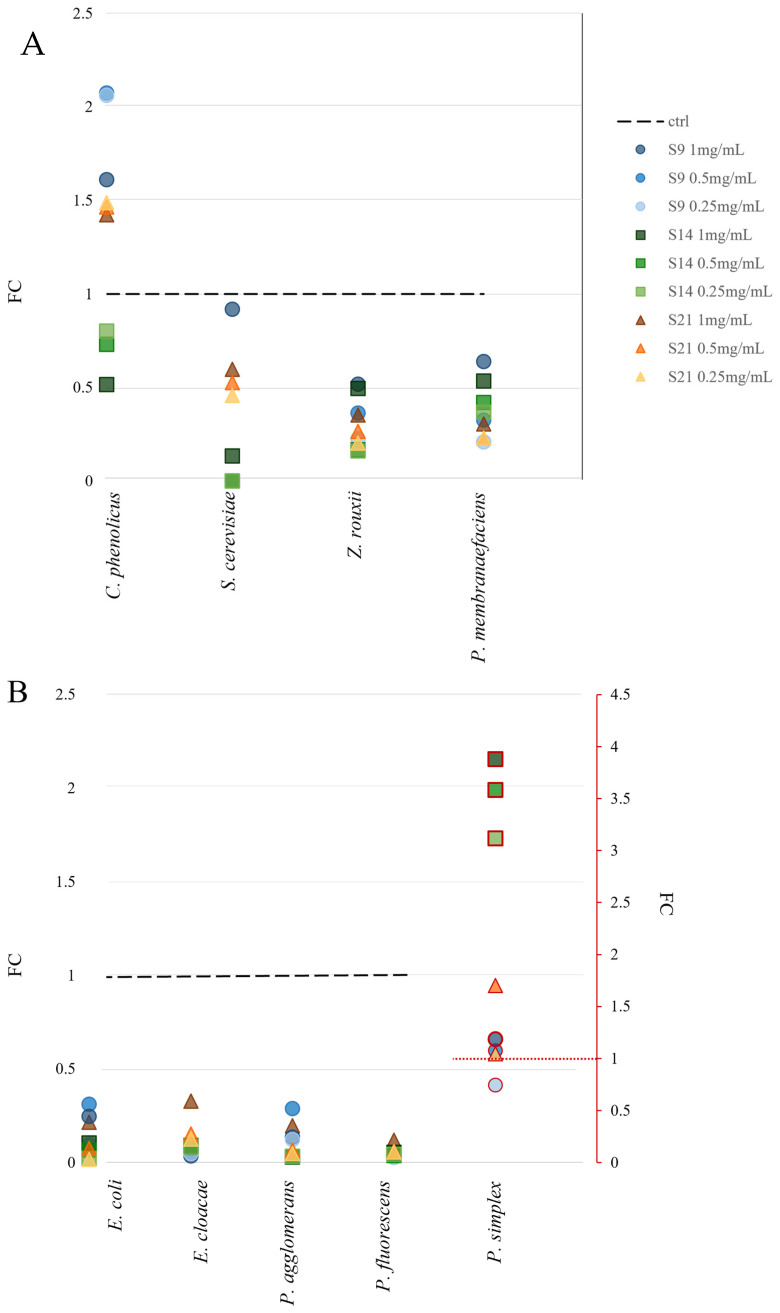
Comparison between single growth of strains in the presence of hemp extracts and in standard conditions, reported as growth percentage. Fold change (*y*-axis) correlates with the growth of the strain under standard conditions, represented by the dashed line. The shapes over this line represent conditions where growth in the extracts exceeded that in the standard medium. Shapes and colours refer to the extracts S9 (blue circle), S14 (green square), and S21 (brown triangle). The colour intensity refers to the concentration tested—the darker the shape, the higher the concentration. Growths were compared in pairs with the *t*-test analysis. All comparisons showed a statistical significance of *p* < 0.05. (**A**) Growth of fungal strains. (**B**) Growth of bacterial strains. For a correct visualization of the results, the growth of *Peribacillus simplex* (red outlined shape) is reported in the right secondary axis (red), due to the constant difference detected in growth values compared to the other strains.

Hemp extracts were tested for their potential use as a source of nutrients. For this reason, four yeast strains and five bacterial strains were incubated with the extracts and 2% of glucose. The growth in such conditions was followed with spectrophotometric techniques and then compared with the control represented by the same strains grown in standard media. The results mainly showed that there is a difference in the response of eukaryotes and prokaryotes. In fact, the fold change compared to the control was over 0.5 for fungi and around 0.3 for bacteria. Fungi demonstrated a preference for S9 (extract after 1 month of maceration), achieving the best results at the concentration of 1 mg mL^−1^ (Figure 6A). Under these conditions, *S. cerevisiae* reached a cellular density of 0.90 compared to when under standard conditions, while *Zygosaccharomyces rouxii* stood at 0.51 and *Pichia membranifaciens* reached 0.62 in growth compared to the control. Interestingly, *Cryptococcus phenolicus* grew better in hemp extracts S9 and S21 than in the standard medium, doubling its growth with respect to the control with S9 at a concentration of 0.5 mg mL^−1^. Overall, prokaryotes registered a value of growth with the extracts much lower than those in standard media. For example, *Pseudomonas fluorescens* did not achieve over 0.12, while *Pantoea agglomerans* and *Enterobacter cloacae* reached, respectively, 0.29 and 0.3 of the control. *Peribacillus simplex* represented an exception because it grew better in the extracts than in the medium reaching a growth that was 4 times the control. Bacteria also exhibited a different inclination towards the extracts in fact, and while *Escherichia coli* and *Pantoea agglomerans* had the best results with S9 at a concentration of 0.5 mg mL^−1^, *E. cloacae* grew well with 1 mg mL^−1^ of S21, and *B. simplex* preferred S14.

## 4. Discussion

Hemp can be considered a “multi-purpose material” because it finds applications in several different fields such as apparel, fabrics, paper, cordage, and building materials [55]. Other than its traditional usage, hemp could also be used for animal feeding, cosmetic and nutraceutical products, or in energy and fuel production. Such a versatile plant produces considerable leftover biomass that could be recovered and transformed into high-value products. Hemp residues in fact contain basic plant structural components (cellulose, hemicellulose, and lignin) that could be converted into bioplastic materials [56], sustainable foams [57], and other biodegradable cost-efficient bio-composites [58]. Nevertheless, hemp residues are also rich in important antioxidant compounds, such as phenolic compounds, flavonoids, and terpenes [59]. In this scenario, the challenge is to develop efficient extraction methods that reduce the consumption of energy and chemicals while maximizing the recovery yield [60]. Conventional extraction methods, in fact, use organic solvents to separate molecules based on the solubility difference of a solute in two immiscible liquid phases [61]. It has been calculated that the use of solvents corresponds to around 80 % of chemical waste during the overall synthetic procedure [62]. The massive use of such solvents has raised concerns regarding the impact on the environment and the potential hazards for human health [63,64]. For this reason, novel extraction methods should make use of green technologies that provide an eco-friendly and sustainable alternative [65,66]. Microorganisms could represent a valid solution for this purpose. Recently, it has been demonstrated that cannabis waste can be converted into bio-fertilizer through a semi-anaerobic process by adding specialized lignin-degrading microbial inoculants that are able to transform it into a more bioavailable form within three weeks [67]. As evidenced by a systematic analysis of the literature [68], the interest in bio-organic fertilizer, i.e., the product obtained by the microbial degradation of organic wastes, has increasingly risen in the last decade. Our research deepened the knowledge of bioconversion processes that occur spontaneously, without the use of external inoculants, whose use could subjected to legal requirements [69]. We took advantage of the principles driving the water-retting methodology, in which the presence of moisture and microorganisms within the plant material enables the breakdown of cellular tissues and adhesive substances surrounding the fibres [70]. We collected industrial hemp waste and soaked it in sterile water for three months. Every week, we sampled part of the material that was intended for microbiological and histological analyses. Both cultural-dependent and -independent analyses showed that the prokaryote community remained relatively stable throughout the degradation process, while fungi displayed considerable variability across the different stages. Within the bacterial community, the most abundant genera in terms of reads sequenced were *Ochrobactrum* and *Achromobacter*. Both were known in the literature for their degrading potential [71,72]; for example, the whole-genome sequencing of the latter demonstrated that genes for aminobenzoate, benzoate, and styrene degradation were present [73]. There is a strong relationship between the two genera. In fact, *Ochrobactrum spp.* are considered to derive from the genus *Achromobacter* [74]. Moreover, organisms formerly called CDC group Vd and *Achromobacter* groups A, C, and D were renamed *Ochrobactrum anthropi* [75], and were recently reclassified as being of the genus *Brucella* [76].

The genus *Achromobacter* is widely distributed in natural environments and includes highly divergent species—from human pathogens to plant-associated species [77,78]. In particular, some strains of this genus were isolated from the rhizosphere and were demonstrated to contribute to protect plants from alkaline stress [79]. *Achromobacter* strains were also found to be part of the endophyte community of *Zea mays* L. [80]. The association between this genus and plants corroborated the sequencing results.

Moreover, the community depicted through MinION sequencing is comparable with other studies regarding the microbiota dynamic during the lab-scale water-retting process. Samples were in fact characterized by *Bacillus*, *Brevibacillus*, and *Pseudomonas* as described by Ventorino and colleagues [81]. We also found a considerable number of reads belonging to *Ochrobactrum* and *Stenotrophomnas* that are in line with the results reported by Zhang and colleagues [82]. Based on the genera detected, we divided the process into three moments: early, medium, and late maceration. The late maceration is characterized by fungi like *Thricoderma* and *Arthrographis*. The former was employed in the waste renewable industry because it synthetizes exogenic fibrolytic enzymes (EFEs), which helped fibre digestion [83,84,85,86]. Meanwhile, the second genus is interesting because it produces a laccase that oxidizes a variety of phenolic substrates [85]. Intriguingly, hierarchical analysis, based on metabarcoding data, clustered the fraction taken after three weeks of maceration together with the late-maceration fractions to indicate, probably, that three weeks could be the first turnaround point in the maceration process. This evidence is supported by the observation reported by Ventorino and colleagues, who registered a strong increase in pectinase activity from 14 to 21 days in all water-retting conditions [81]. The medium-maceration stage (from the fourth to sixth weeks of degradation) is characterized by a high abundance of *Saccharomyces* and *Nakasomyces* reads. This result matched a peak of eukaryotic cell density registered at t4 with the cultural analysis. Such evidence could be considered a sign of the presence of an assimilable carbon source, which could derive from the first three weeks of degradation. An interesting result is that the fraction t12, taken after three months of maceration, is comparable to the medium-stage fractions both from cultural-dependent and -independent analyses. It is in fact characterized by a huge increase in yeast cell density and the presence of *Nakasomyces* reads. A second turnaround in the maceration process could be established after two months of degradation. Histological analysis also confirmed that, although signs of degradation were present in the chloroplasts after just one month of maceration, the actual cellular damage was only observable after two and three months of maceration. The other point of this study was to demonstrate the feasibility of extracting useful compounds from hemp residues. We performed a comparison among extracts after three days and one and two months of maceration. All the techniques employed to characterize hemp extracts demonstrated that one-month extract is the richest in carbohydrates, amino acids, and bioactive compounds. On the contrary, in the short-term extract, the level of degradation was not sufficient to have assimilable compounds, and the pH of the environment where hemp was macerating limited the solubility of proteins and thus their concentration in the final product. Conversely, the extract after two months was the best in terms of the amount of amino acids, but it lost the major part of the bioactive compounds, including carbohydrates, that could have been metabolized by yeasts, as described before. The presence of compounds such as choline, trigonelline, and GABA in the one-month extract is noteworthy. The first, in fact, is an essential nutrient for humans that needs to be assumed through the diet [87]. Meanwhile, trigonelline can be employed in the treatment of diabetes and central nervous system diseases [88,89]. Similarly, GABA is central to many physiological functions, such as anti-hypertensive and antidepressant activities [90].

Considering the heterogeneity of such molecules, HEs could find application in many biotechnological fields, such as antibiofilm compounds [36] and bio-organic fertilizers in sustainable agriculture or animal feeding [91]. The growth assays demonstrated that HEs could positively interact with yeasts, while they had a limited beneficial effect on bacteria.

## 5. Conclusions

Our data described in detail the dynamics of the microbial population during the bioconversion of hemp residues and demonstrated that the endogenous microbial population can accomplish the degradation of waste spontaneously using water as the only solvent. One month is the time needed to achieve an extract that has the best composition in terms of nutrients and bioactive compounds. Such an extract could sustain the growth of yeast cells in the absence of other nitrogen sources. The microbial pretreatment of biomass turned out to be a simple, effortless, and cost-effective method for extracting valuable compounds from waste material. Such molecules could be recycled nutrients within the food supply chain and also helpful dietary supplements for human health.

## Figures and Tables

**Table 1:** Hemp extracts (HEs) obtained by mechanical pressing at the end of the short- and long-term maceration process in water.

Sample ID	Plant Variety	Waste Material	Maceration Time	Maceration Method
S14	*Cannabis sativa* L. cv. Strawberry	Dry flowers and leaves	72 h	Short-term
S9	*Cannabis sativa* L. cv. Strawberry	Dry flowers and leaves	1 month	Long-term
S21	*Cannabis sativa* L. cv. Strawberry	Dry flowers and leaves	2 months	Long-term

Legend. HE S14 was obtained with short maceration and protein precipitation in addition to the mechanical extraction method. HEs S9 and S21 were obtained using mechanical extraction after one and two months of water maceration, respectively, without any pretreatment before the extraction.

**Table 2:** Strains employed for the growth assay. All strains were provided by the CMC collection of CEMIN.

SPECIES	Collection Id	Source of Isolation
*Cryptococcus phenolicus*	CMC 1668	Soil
*Pichia membranaefaciens*	CMC 206	Soil
*Saccharomyces cerevisiae*	CMC 207	CBS Type strain
*Zygosaccharomyces rouxii*	CMC 259	Soil
*Peribacillus simplex*	CMC 85	Seed
*Enterobacter cloacae*	B 89	Seed
*Escherichia coli*	B 6	Faecis
*Pantoea agglomerans*	B 35	Seed
*Pseudomonas fluorescens*	B 57	Seed

## Data Availability

The original contributions presented in this study are included in the article/Supplementary Material. Further inquiries can be directed to the corresponding authors.

## Supplementary Materials

The following supporting information can be downloaded at: https://www.mdpi.com/article/10.3390/microorganisms13020455/s1, Figure S1: Hierarical clustering of fractions.
