# Supplementary material for: Spontaneous Bio-Recycling: Recovering Bioactive Molecules Through Endogenous Microbial Maceration of Hemp Residues
Source: Microorganisms. 2025 Feb 19;13(2):455. doi: 10.3390/microorganisms13020455 (PMC11858476; doi:10.3390/microorganisms13020455)

Supplementary Figure S1 Hierarical clustering of fractions. Heatmap showing the reads detected for each taxa (per rows) in each fraction sampled during maceration process (columns). The intensity of the rectangle colour indicates the relative abundance of a specific taxa compared to the other included in the same fraction: white rectangle means low intensity while blue rectangle represents a high number of reads detected. A dendrogram is used to visualize results from clustering algorithm. Fractions t0, t1 and t2 (green block) form a separate cluster. t3, t7 and t8 (blue block) are grouped together indicating a good similarity. The last right-most part of the dendrogram shows fractions clustered into two couples (t12-t4 and t6-t5). Clustering algorithm confirmed that fractions can be divided into three groups: early maceration (t0, t1, t2), medium maceration (t4, t5, t6 and t12) and late maceration (t7, t8 and t3)

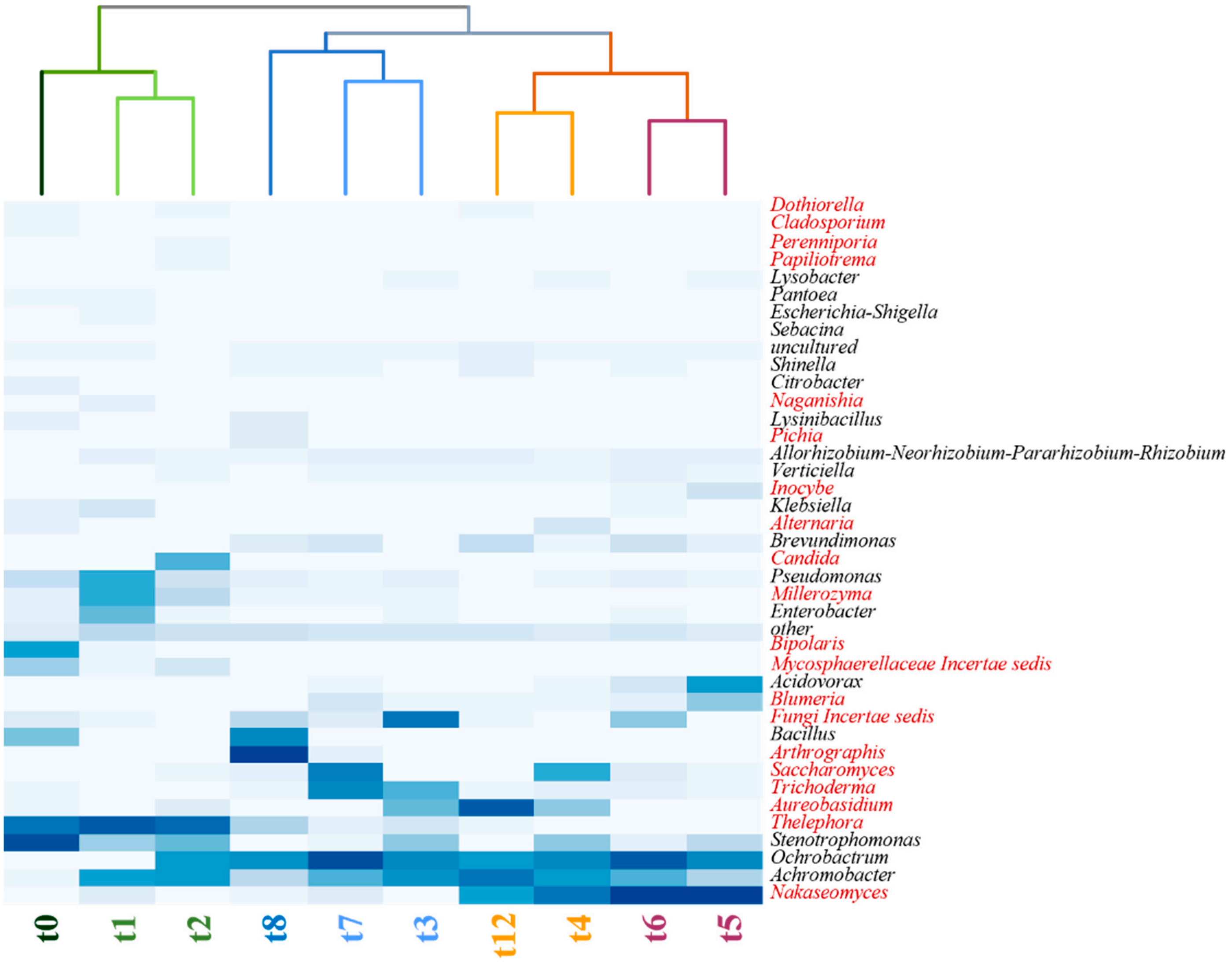

Supplement: Supplementary file 1 [file microorganisms-13-00455-s001.zip › microorganisms-3423989-supplementary.pdf]
